# Optimal cut-off points for waist circumference in the definition of metabolic syndrome in Brazilian adults: baseline analyses of the Longitudinal Study of Adult Health (ELSA-Brasil)

**DOI:** 10.1186/s13098-018-0347-0

**Published:** 2018-06-15

**Authors:** Thiane Ristow Cardinal, Alvaro Vigo, Bruce Bartholow Duncan, Sheila Maria Alvim Matos, Maria de Jesus Mendes da Fonseca, Sandhi Maria Barreto, Maria Inês Schmidt

**Affiliations:** 1 Postgraduate Program in Epidemiology, School of Medicine, Federal University of Rio Grande do Sul, Rua São Luis 662/1405, Porto Alegre, Rio Grande do Sul CEP 90620-170 Brazil; 2 Postgraduate Program in Epidemiology, School of Medicine, Federal University of Rio Grande do Sul, Rua Ramiro Barcelos, n◦ 2600, sala 519, Porto Alegre, Rio Grande do Sul CEP 90035-003 Brazil; 3 Department of Statistics, Federal University do Rio Grande do Sul, Rua Ramiro Barcelos, n◦ 2600, sala 519, Porto Alegre, Rio Grande do Sul CEP 90035-003 Brazil; 4 Postgraduate Program in Epidemiology, School of Medicine, Federal University of Rio Grande do Sul, Rua Ramiro Barcelos, n◦ 2600, sala 519, Porto Alegre, Rio Grande do Sul CEP 90035-003 Brazil; 5 Department of Social Medicine, School of Medicine, Federal University of Rio Grande do Sul, Rua Ramiro Barcelos, n◦ 2600, sala 519, Porto Alegre, Rio Grande do Sul CEP 90035-003 Brazil; 6 Institute of Collective Health, Federal University of Bahia, Rua Basílio da Gama, Canela, Salvador, Bahia CEP 40000-110 Brazil; 7 Departament of Epidemiology and Quantitative Methods in Health, National School of Public Health FIOCRUZ, Rua Leopoldo Bulhões 1480, Sala 818, Rio de Janeiro, Rio de Janeiro CEP 21041-210 Brazil; 8 Research Group on Epidemiology on Chronic and Occupational Diseases (GERMINAL), School of Medicine, Universidade Federal de Minas Gerais, Avenida Alfredo Balena 190, sala 814, Belo Horizonte, Minas Gerais CEP 30130100 Brazil

**Keywords:** Waist circumference, Metabolic syndrome, Cut-off point

## Abstract

**Background:**

Waist circumference (WC) has been incorporated in the definition of the metabolic syndrome (MetS) but the exact WC cut-off points across populations are not clear. The Joint Interim Statement (JIS) suggested possible cut-offs to different populations and ethnic groups. However, the adequacy of these cut-offs to Brazilian adults has been scarcely investigated. The objective of the study is to evaluate possible WC thresholds to be used in the definition of MetS using data from the Longitudinal Study of Adult Health (ELSA-Brasil), a multicenter cohort study of civil servants (35–74 years old) of six Brazilian cities.

**Methods:**

We analyzed baseline data from 14,893 participants (6772 men and 8121 women). A MetS was defined according to the JIS criteria, but excluding WC and thus requiring 2 of the 4 remaining elements. We used restricted cubic spline regression to graph the relationship between WC and MetS. We identified optimal cut-off points which maximized joint sensitivity and specificity (Youden’s index) from Receiver Operator Characteristic Curves. We also estimated the C-statistics using logistic regression.

**Results:**

We found no apparent threshold for WC in restricted cubic spline plots. Optimal cut-off for men was 92 cm (2 cm lower than that recommended by JIS for Caucasian/Europids or Sub-Saharan African men), but 2 cm higher than that recommended for ethnic Central and South American. For women, optimal cut-off was 86, 6 cm higher than that recommended for Caucasian/Europids and ethnic Central and South American. Optimal cut-offs did not very across age groups and most common race/color categories (except for Asian men, 87 cm).

**Conclusions:**

Sex-specific cut-offs for WC recommended by JIS differ from optimal cut-offs we found for adult men and women of Brazil´s most common ethnic groups.

## Background

The metabolic syndrome (MetS), a multiplex risk factor for atherosclerotic cardiovascular disease and type 2 diabetes mellitus [1], is now common throughout the world [2]. Observed in clinical practice for decades, the MetS is now recognized as having public health importance, most probably due to the growing global prevalence of obesity [3].

The inclusion of waist circumference (WC) in the definition of MetS is now supported by major bodies, although a single cut-off point for international use is not possible owed to its wide variation across populations [4]. This led the International Diabetes Federation [5] to propose population-specific thresholds for WC, a position which was endorsed by the World Health Organization [6]. The Joint Interim Statement (JIS) suggested WC cut-offs for different ethnic groups based on the literature and consensus [7].

Given the ethnic diversity and admixture of the Brazilian population, the application of WC cut-offs recommended by JIS is not straightforward. Specifically, whether values recommended for white Caucasians/Europids or black Sub-Saharan Africans (≥ 94 cm for men and ≥ 80 cm for women) are applicable to similar Brazilian adults is questionable. Additionally, cut-offs referred for ethnic Central and South American ethnic groups (≥ 90 cm for men and ≥ 80 cm for women) are unlikely to be useful to the specific Brazilian ethnic groups.

Thus, adequacy of optimal WC cut-off to define MetS needs further evaluation in Brazil. The Brazilian Longitudinal Study of Adult Health (ELSA-Brasil) permits such analyses in a large number of men and women of a broad age range (35–74 years), of different ethnic groups, living and working in different cities of Brazil. The aim of this cross-sectional study is to evaluate possible cut-off points for WC for men and women against the presence of MetS. We will also explore the application of these values for different age and ethnic groups.

## Methods

ELSA-Brasil is a cohort study of 15,105 civil servants aged 35–74 years, enrolled at six universities or research institutes located in three different regions of Brazil: the Federal Universities of Bahia, Espirito Santo, Minas Gerais, and Rio Grande do Sul; the University of São Paulo; and the Oswaldo Cruz Foundation. A detailed protocol for the Study can be found else where [8, 9]. Study approval was obtained from the institutional review boards of all the centers, and all subjects signed an informed consent form.

We used baseline data after performing the following 212 exclusions: three participants who did not have complete data on WC, 174 on self-declared race/colour, and 35 on other information needed do define the MetS. The final sample was composed of 14,893 participants (6772 men and 8121 women).

After an overnight fast of 12 h we performed anthropometric and blood pressure measurements according to standard protocols [10–12], and then collected venous blood.

WC was measured using an inelastic tape of 150 cm (Mabis-Gulick, Waukegan, IL, USA) at the midpoint between the lowest rib margin and the iliac crest. The intra-class correlation coefficient for repeat measurements was 0.98 (95% CI 0.85–1.0) [11]. We measured height to the nearest 0.1 cm (Seca-SE-216, Hamburg, Germany). We measured body weight with an electronic scale with maximum capacity of 200 kg (Toledo, São Bernardo do Campo, Brazil). Body mass index (BMI) was calculated by dividing weight (kg) per height squared (m^2^).

Blood pressure was taken using an oscillometric device (Omron HEM 705CPINT) after a 5-min rest with the subject in a sitting position in a quiet, temperature-controlled room (20–24 °C). Three measurements were taken at 2-min intervals and the mean of the last two was used for these analyses. Intra-class correlation coefficients for systolic and diastolic blood pressure were 0.88 (95% CI 0.82–0.91) and 0.89 (95% CI 0.83–0.92), respectively [11].

We determined plasma glucose enzymatically by the hexokinase method (ADVIA Chemistry; Siemens, Deerfield, Illinois). High-density lipoprotein cholesterol (HDL-C), and triglycerides were determined by the enzymatic colorimetric method (ADVIA Química) [13]. Blinded quality control analyses revealed intra-class correlation coefficients for glucose, HDL-C and triglycerides of 0.99 (95% CI 0.95–1.00), 0.97 (95% CI 0.95–0.98) and 1.0 (95% CI 0.99–1.00), respectively [14].

We defined components of MetS according to JIS [7]: elevated systolic blood pressure (≥ 130 mmHg) or diastolic blood pressure (≥ 85 mmHg) or use of antihypertensive medication; high triglycerides as ≥ 150 mg/dL or use of lipid-lowering agents (fibrate and nicotinic acid); low HDL-C as < 40 mg/dL for men and < 50 mg/dL for women, or use of specific medication (fibrate and nicotinic acid); high fasting plasma glucose as ≥ 100 mg/dL or use of antidiabetic medication; WC as population-specific cut-offs. For the analyses here reported, we excluded WC from the definition of MetS, which then was defined as requiring the presence of at least two out of four components.

Sociodemographic variables were obtained by structured interviews while participants remained at the clinic center and included age, gender, education level, self-declared race. Medication use was verified against prescription, package or package inserts.

The relationship between WC and MetS was initially investigated with logistic regression using restricted cubic splines. Four “knots” were positioned in percentiles 10, 25, 75 and 90 of the empirical distribution of WC. The polynomial expansions were created by macros DASPLINE and DSHIDE (developed by Frank E Harrell Jr) for SAS (Statistical Analysis System). To test the linearity of the relationship between WC and the logit of the probability of having MetS we used the Wald statistics. The model included terms for age, sex and for the interaction between WC and sex. We used the value defining the first quartile of the sex-specific WC distribution as a reference for the estimation of odds ratios.

The probability that a randomly chosen subject with the MetS is more likely to be rated as such than a randomly chosen subject without the MetS was estimated by the total area under the curve (AUC) and its respective 95% confidence intervals using Receiver Operator Characteristics (ROC) [15] in logistic regression (SPSS—Statistical Package for the Social Sciences, version 18 for Windows). Optimal values for WC, assuming equal an importance for the sensitivity and specificity of the cut-off point were defined by the Youden index [maximum (sensitivity + specificity − 1)]. We also estimated sensitivity, specificity, positive and negative predictive values, impact on MetS prevalence, and relative increase in the odds of MetS (odds ratio, OR). Significance level was set at 5%. Unless otherwise specified, all analyses were carried out using SAS 9.4 (SAS Institute, Inc., Cary, North Carolina). Given the well-established use of sex-specific thresholds for WC, all analyzes were performed separately for men and women.

## Results

Among the 14,893 participants in the ELSA-Brasil, 54.5% (8121) were women. Table 1 shows that, in men and woman, most individuals were between 45 and 64 years (67.3%) and 52.2% declared being white. About 87% had completed at least high school. Around 63% of the participants were overweight (BMI ≥ 25 kg/m^2^). Prevalence of individual components of MetS when defined by the JIS was higher in men, except for elevated WC (based on JIS cut-offs for Central and South American ethnic groups and white Caucasians/Europids or black Sub-Saharan Africans) and low levels of HDL-C (Table 1).

**Table 1:** Characteristics of 14,893 participants in the Brazilian Longitudinal Study of Adult Health (ELSA-Brasil), 2008–2010

Characteristics	Total sample		Men		Women	
	n	%	n	%	n	%
Age (years)						
35–44	3312	22.2	1543	22.8	1769	21.8
45–54	5867	39.4	2644	39.0	3223	39.7
55–64	4157	27.9	1816	26.8	2341	28.8
65–74	1557	10.5	769	11.4	788	9.7
Self-declared skin colour/race category						
White	7771	52.2	3589	53.0	4182	51.5
Brown (‘pardo’)	4197	28.2	2023	29.9	2174	26.8
Black	2394	16.1	940	13.9	1454	17.9
Asian	374	2.5	128	1.9	246	3.0
Indigenous	157	1.0	92	1.3	65	0.8
Educational level						
Incomplete primary education	876	5.9	551	8.2	325	4.0
Complete primary education	1019	6.8	570	8.4	449	5.5
Complete high school	5179	34.8	2250	33.2	2929	36.1
University degree	7819	52.5	3401	50.2	4418	54.4
Body mass index						
Low/normal (BMI < 25 kg/m^2^)	5494	36.9	2312	34.2	3182	39.2
Overweight (25 ≤ BMI < 30 kg/m^2^)	5995	40.3	3063	45.2	2932	36.1
Obesity (BMI ≥ 30 kg/m^2^)	3398	22.8	1394	20.6	2004	24.7
Metabolic syndrome^a^						
If WC defined by JIS for ethnic Central and South Americans^b^	6960	46.7	3663	54.1	3297	40.6
If WC defined by JIS for Caucasians/Europids or Sub-Saharan Africans^c^	6587	44.2	3290	48.6	3297	40.6
Components of metabolic syndrome^a^						
Elevated WC						
Defined for ethnic Central and South Americans^b^	10,312	69.2	4518	66.7	5794	71.3
Defined for Caucasians/Europids or Sub-Saharan Africans^c^	9365	62.9	3571	52.7	5794	71.3
Elevated fasting glucose^d^	10,664	71.6	5525	81.6	5139	63.3
Elevated blood pressure^e^	7637	51.3	4040	59.7	3597	44.3
Elevated triglycerides^f^	4705	31.6	2806	41.4	1899	23.4
Low HDL-C^g^	2807	18.8	1086	16.0	1721	21.2

*WC* waist circumference, *HDL-C* high density lipoprotein cholesterol, *JIS* Joint Interim Statement [7]

^a^ Defined by the JIS criteria, using specific WC cut-offs

^b^ WC ≥ 90 cm for men; ≥ 80 cm for women

^c^ WC ≥ 94 cm for men; ≥ 80 cm for women

^d^ Fasting glucose ≥ 100 mg/dL or drug treatment for elevated glucose

^e^ Systolic ≥ 130 mmHg and/or diastolic ≥ 80 mmHg, or antihypertensive drug treatment

^f^ Triglycerides ≥ 1.69 mmol/L (150 mg/dL) or drug treatment for elevated triglycerides (fibrates and nicotinic acid)

^g^ HDL-C < 1.03 mmol/L (40 mg/dL) for men and < 1.29 mmol/L (50 mg/dL) for women, or drug treatment for low HDL-C (fibrates and nicotinic acid)

**Fig. 1:**
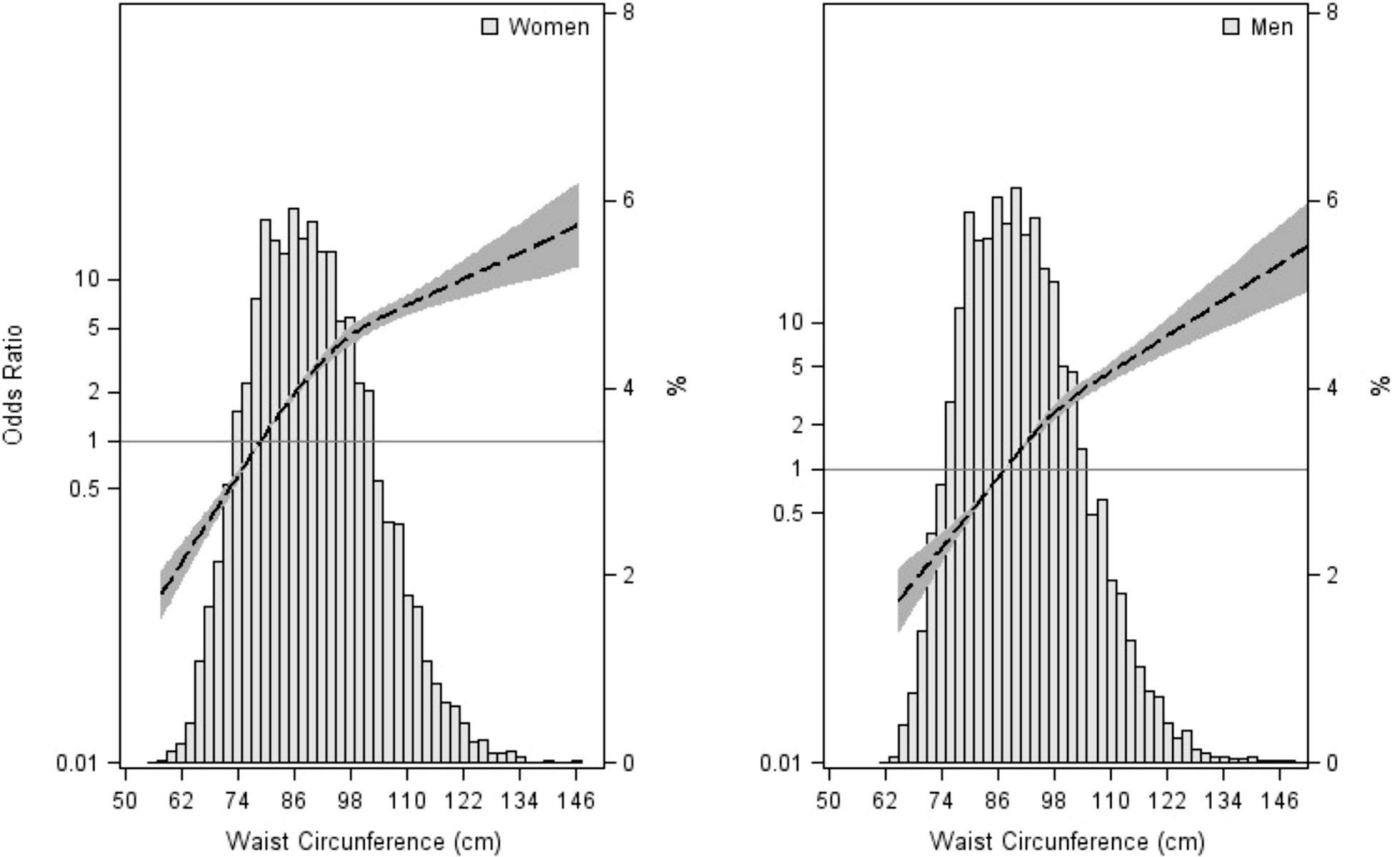
Restricted cubic spline graphical analysis, showing the odds ratios for waist circumference. The dashed line shows odds ratios, and their upper and lower 95% confidence intervals are shown in grey. Reference category for WC was defined by the first quartile value for men (< 87.5 cm) and women (< 78 cm)

In curves derived from cubic spline regression models, the logit of the probability of having the MetS (WC not included in the definition) increased continuously with increasing values of WC, although at very high values the rate of increase slowed down (data not shown). We found a similar pattern while plotting WC values against odds ratios for having MetS, taking the value of the first quartile of gender-specific WC distributions (87.5 cm for men and 78.0 cm for women) as a reference group (Fig. 1).

**Fig. 2:**
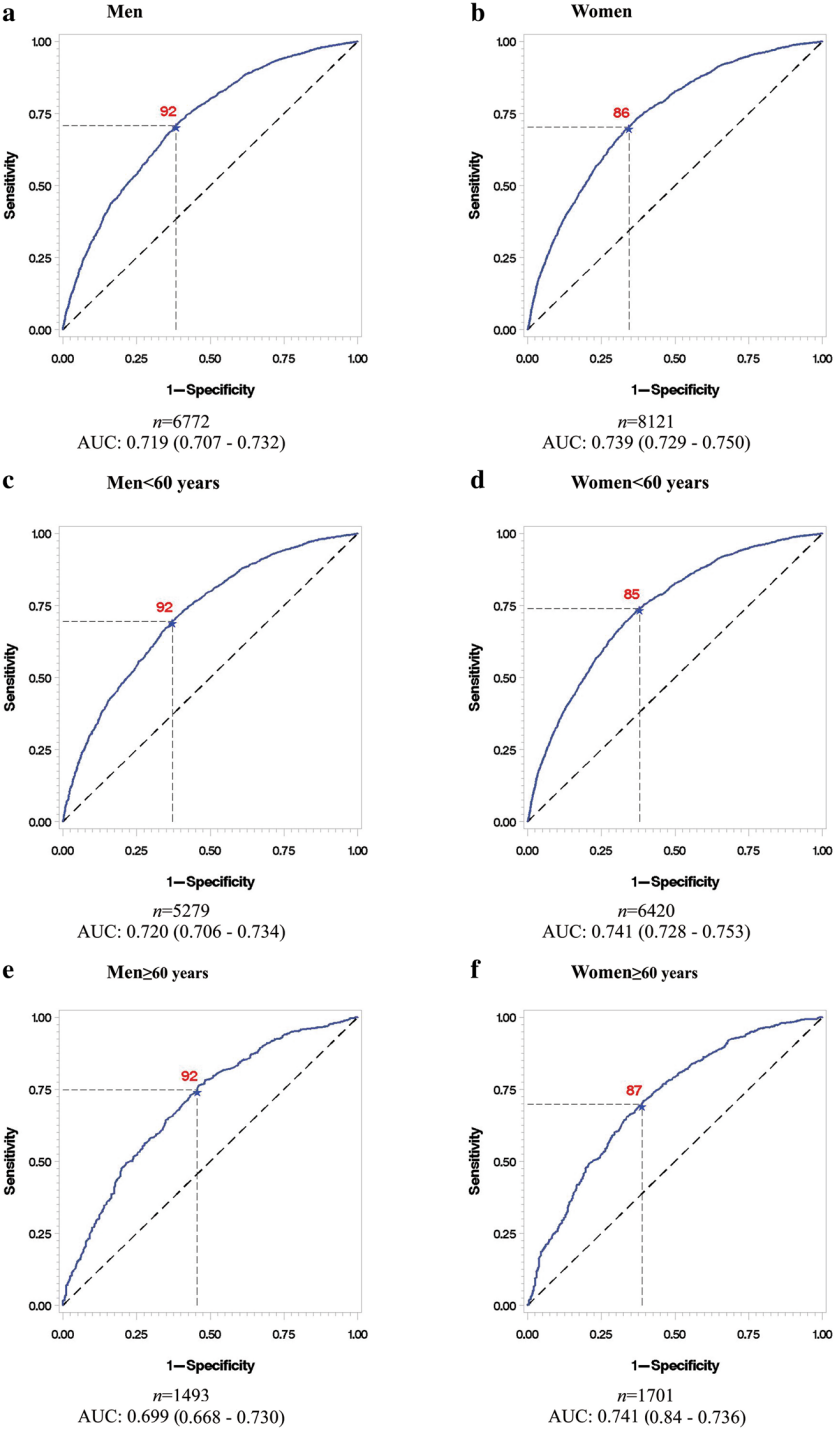
ROC analyses for waist circumference in the definition of MetS by sex and age. Top panel (**a** and **b**): overall sample. Lower panels (**c**, **d**, **e** and **f**): sex/age specific analyses. Area under the curve (AUC)

Thus, in the absence of a natural threshold for WC, we explored optimal cut-offs by examining sensitivity and 1-specificity for having MetS in ROC curves across the spectrum of WC (Fig. 2). The total area under the curve (AUC) was 0.719 (95% CI 0.707–0.732) for men, and 0.739 (95% CI 0.729–0.750) for women (Fig. 2a and b). The optimal cut-off of WC for men was 92 cm (Fig. 2a) and for women was 86 cm (Fig. 2b). Also seen in Fig. 2c and d, optimal cut-offs for those under 60 years were close to those for 60 years and over, although among women, a slightly higher cut-off was seen for those over 60 years.

**Fig. 3:**
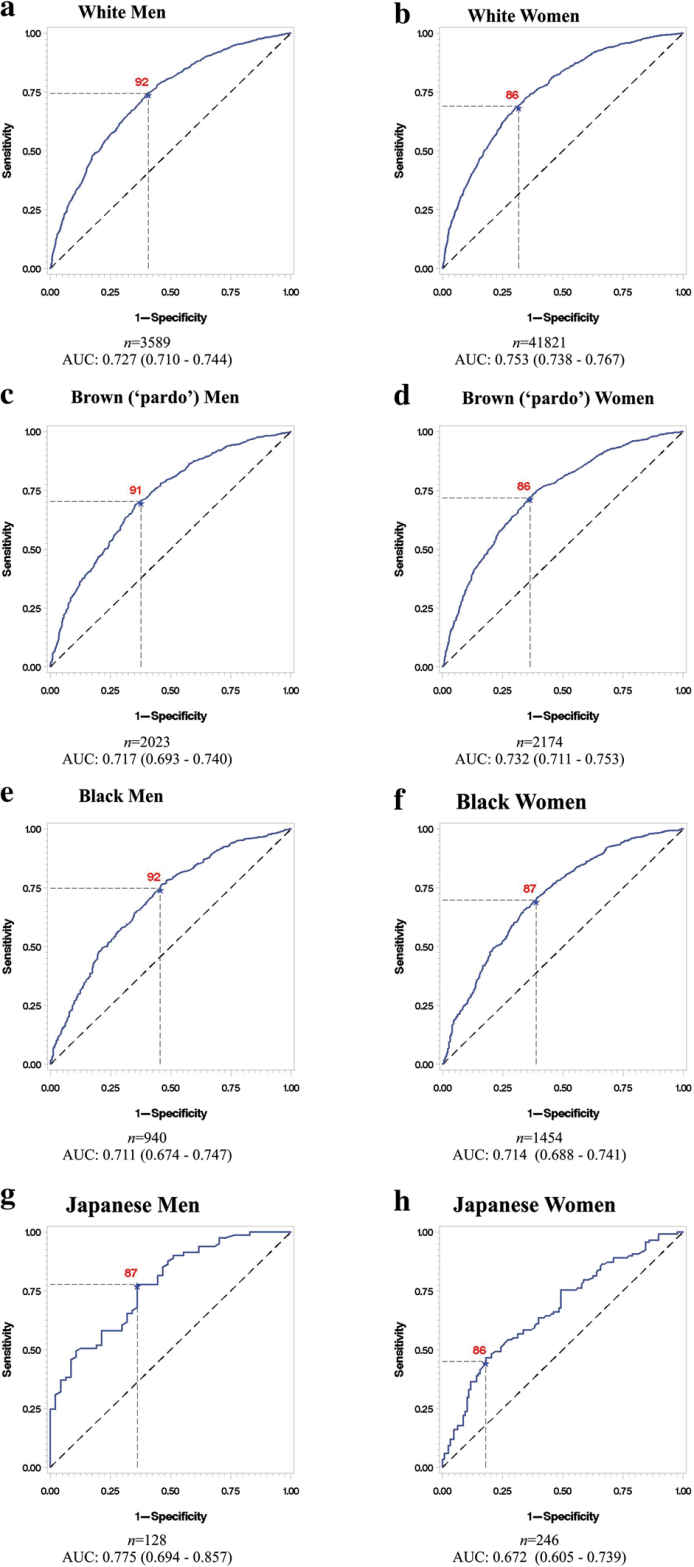
ROC analyses for optimal waist circumference cut-off in the definition of MetS according ethnic groups. Area under the curve (AUC). *Indigenous group not shown because of insufficient sample size. **a** Men; **b** women; **c** men <60 years; **d** women <60 years; **e** men >=60 years; **f** women >=60 years

Optimal cut-offs were also similar across Brazilian ethnic groups (Fig. 3), although a few observations can be made. Among women, we noted a slightly lower optimal cut-off for blacks (84 vs 86 cm for all women). Among men we noted a lower optimal cut-off for Asians (87 vs 92 cm for all men). Our sample size was insufficient to estimate optimal cut-offs the indigenous subjects, the AUC for indigenous men being 0.677 (95% CI 0.561–0.793) and for indigenous women, 0.628 (95% CI 0.484–0.773) (data not shown).

Table 2 summarizes diagnostic properties for these optimal cut-offs (in italic) and for additional ones considered within a clinically relevant range. As indicated by the odds ratios, these possible cut-offs were associated with increased probability of having the MetS. Of clinical relevance, prevalence of MetS was influenced by the WC cut-off used, varying from 54.1 to 38.4% among men, and from 40.5 to 31.6% among women.

**Table 2:** Cut-off points for waist circumference (WC) in the definition of metabolic syndrome as recommended by the Joint Interim Statement criteria (not including WC)

WC	Sens. %	Spec.%	PPV %	NPV %	Youden	Prevalence MetS^c^ (%)	Odds ratio^d^	
							Value	95% CI
Men								
90^a^	77.9	53.3	74.9	57.4	0.3119	54.1	1.26	1.22–1.30
91	74.8	57.6	76.0	56.0	0.3241	52.9	1.38	1.32–1.44
*92*	*71.2*	*61.2*	*76.7*	*54.3*	*0.3245*	*51.5*	*1.50*	*1.42–1.59*
93	67.7	64.5	77.4	52.7	0.3220	50.1	1.64	1.53–1.76
94^b^	63.9	67.2	77.7	50.9	0.3110	48.6	1.78	1.64–1.93
95	59.7	70.8	78.5	49.5	0.3048	47.0	1.93	1.76–2.12
96	56.0	73.6	79.1	48.3	0.2954	45.7	2.09	1.88–2.32
97	52.2	76.6	80.0	47.2	0.2884	44.3	2.25	2.01–2.52
98	48.4	80.0	81.2	46.4	0.2836	43.0	2.42	2.14–2.73
99	44.8	82.8	82.3	45.5	0.2755	41.8	2.59	2.27–2.94
100	40.9	85.0	83.0	44.5	0.2593	40.4	2.76	2.41–3.15
101	37.9	86.5	83.4	43.8	0.2444	39.6	2.93	2.56–3.35
102	34.3	88.1	83.8	42.8	0.2244	38.4	3.10	2.71–3.55
Women								
80^ab^	87.4	41.9	55.3	80.2	0.2932	40.5	1.11	1.09–1.12
81	84.8	46.4	56.5	78.8	0.3122	39.8	1.22	1.19–1.25
82	82.4	50.3	57.6	77.6	0.3265	39.0	1.34	1.30–1.39
83	79.4	53.9	58.6	76.0	0.3322	38.1	1.48	1.42–1.54
84	77.2	57.6	59.9	75.5	0.3478	37.6	1.62	1.55–1.70
85	74.3	61.4	61.3	74.4	0.3574	36.6	1.77	1.68–1.87
*86*	*70.8*	*65.1*	*62.5*	*73.0*	*0.3589*	*35.6*	*1.93*	*1.83–2.05*
87	67.5	68.2	63.5	71.8	0.3566	34.7	2.10	1.98–2.24
88	64.1	71.1	64.5	70.6	0.3513	33.7	2.28	2.14–2.44
89	60.5	73.6	65.4	69.4	0.3419	32.6	2.47	2.30–2.66
90	57.1	76.2	66.4	68.4	0.3333	31.6	2.67	2.47–2.89

Brazilian Longitudinal Study of Adult Health (ELSA-Brasil), 2008–2010

*WC* waist circumference, *Sens*. sensitivity, *Spec*. specificity, *PPV* positive predictive value, *NPV* negative predictive value, *MetS* metabolic syndrome, *95% CI* 95% confidence interval

^a^ Cut-off suggested by the Joint Interim Statement (JIS) for Central and South Americans ethnic groups

^b^ Cut-off suggested by the JIS for white Caucasians/Europids or black Sub-Saharan African origin

^c^ Prevalence in the overall sex-specific sample

^d^ Relative odds of the metabolic syndrome being present above the cut-off point, reference strata being the lowest 25% of WCs

## Discussion

In this large sample of Brazilian adults, we did not find a threshold indicative of increased risk for MetS. Optimal (Youden) WC cut-off points were 92 cm for men and 86 cm for women. These cut-offs were associated with increased risk of having MetS of 50% among men and of 93% among women. Optimal cut-offs were similar across age groups and most common Brazilian race/color categories. Among women, optimal cut-offs were higher than those recommended by JIS [7] for white Caucasians/Europids or black Sub-Saharan Africans and Central and South American ethnic groups. Among men, optimal cut-offs were lower than those recommended by JIS [7] for whites and higher for those recommended for blacks and Central and South American ethnic groups.

Optimal cut-off points recommended internationally vary widely. A review of 61 studies [16] found values for men ranging between 72.5 and 103 cm, and for women between 65.5 and 101.2 cm. Values suggested in the literature for the Brazilian adults range between 85 and 99 cm for men, and 80 and 87.5 cm for women [17–27]. Although this variation is largely due to different anthropometric characteristics of various populations, the method for WC measurement and the outcome chosen to validate WC cut-off are also important factors to explain large variation in findings across studies.

Two Brazilian studies using outcomes resembling the MetS found optimal cut-offs closer to those we found. A study conducted in the city of Pelotas, Rio Grande do Sul, using a WC measurement similar to ours and using cardiovascular risk (previous medical diagnosis of hypertension, diabetes mellitus and/or dyslipidemia) as the outcome, found values 95 cm for men, and 87 cm for women [21]. Another study using coronary risk (systolic and diastolic blood pressure, total cholesterol, HDL-C, cigarette smoking and diabetes) conducted in the state of Bahia [26], investigating only women, also found as a result the optimal cut-off point for the WC of 86 cm.

Thus, our findings, supported by these two other Brazilian studies [21, 26], question the application to Brazilian adults, of the cut-offs recommended by JIS define MetS into white, black, Central and South Americans ethnic groups. It is possible that the anthropomorphic characteristics of whites and specific ethnic groups living in Brazil differ from the Caucasians/Europids whites living in Europe, or the blacks of Sub-Saharan Africans and the ethnic groups implied in the JIS report for Central and South America. This might be the case also for Mexican adults, since the cut-offs recommended for men (98 cm) [28] and women (96.6 cm) [29] are much higher than the ones proposed by JIS.

Of note also, when we investigated optimal cut-offs for distinct ethnic groups living in Brazil we did not find important differences, except for those of Asian origin, particularly for men. These findings are in agreement with their generally described lower cut-offs for obesity/central obesity [5, 30–32]. The similarity we found for optimal cut-offs for whites, blacks and mixed race/color groups in Brazil contrasts with findings of Zhu et al. [33] based on The Third National Health and Nutrition Examination Survey (NHANES III) for US adults. Although for women they found only small differences in optimal cut-offs across ethnic groups, among men, whites had 5–6 cm higher cut-off points than blacks, and Mexican Americans had intermediate values.

Which values should be used to define an elevated WC has not been established for the Brazilian populations. Our findings do not support the recommendations suggested in the JIS. For example, the cut-off of 80 cm for women had very low specificity (41.9%). Although the definition of an elevated WC as a component of the MetS is challenging [7], we could suggest values for Brazilian adults close to 92 cm for men and 86 cm for women. These values are applicable to whites, blacks, and mixed race-color groups, although for men of Asian origin (mostly of Japanese ancestry) perhaps slightly lower cut-offs could be used, which is consistent with the JIS recommendations. We did not have enough sample size to evaluate cut-offs for indigenous groups living in Brazil. Additionally, within the age range of our study (35–74 years), we did not find WC differences with age suggesting the need to establish specific cut-offs for specific age groups.

Despite the limitations of a cross-sectional study of this nature, our analyses have some strengths. First, measurements of WC and MetS were highly standardized. Second, the sample size for whites, blacks and mixed color groups allowed specific evaluation of the most common ethnic groups in living in Brazil. Third, the wide age range allowed evaluation of cut-offs for adults and the elderly.

We believe that the choice of the Youden Index to define the optimal cut-off point is adequate, as the MetS has been described as a clustering of risk factors. The Youden index detects the cut-off point which identifies maximum sensitivity and specificity of clusters (2 or more) of the other MetS factors being present when suggested by a high WC value and being absent when suggested by a low WC value. As such, it maximizes the correct identification of clusters.

## Conclusions

In conclusion, sex-specific cut-offs for WC we found for white and black Brazilian adult men (92 cm) and women (86 cm) are 2 cm lower and 6 cm higher, respectively, than those recommended by JIS. Consistent with the literature, the WC cut-off for Brazilian men of Japanese ancestry was lower. Optimal WC cut-offs identified in ELSA-Brasil can be applied to the definition of MetS for Brazilian adults and the elderly.

## Abbreviations

AUC: area under the curve
BMI: body mass index
ELSA-Brasil: The Brazilian Longitudinal Study of Adult Health
HDL-C: high-density lipoprotein cholesterol
JIS: Joint Interim Statement
MetS: metabolic syndrome
NHANES III: The Third National Health and Nutrition Examination Survey
OR: odds ratio
ROC: Receiver Operator Characteristics
SAS: Statistical Analysis System
SPSS: Statistical Package for the Social Sciences
WC: waist circumference
